# A stimulus exposure of 50 ms elicits the uncanny valley effect

**DOI:** 10.1016/j.heliyon.2024.e27977

**Published:** 2024-03-12

**Authors:** Jodie Yam, Tingchen Gong, Hong Xu

**Affiliations:** a Psychology, School of Social Sciences, Nanyang Technological University, Singapore; b Department of Neuroscience, Physiology and Pharmacology, University College London, UK

**Keywords:** Categorical uncertainty, Eeriness, Face perception, Uncanny valley, Visual processing

## Abstract

The uncanny valley (UV) effect captures the observation that artificial entities with near-human appearances tend to create feelings of eeriness. Researchers have proposed many hypotheses to explain the UV effect, but the visual processing mechanisms of the UV have yet to be fully understood. In the present study, we examined if the UV effect is as accessible in brief stimulus exposures compared to long stimulus exposures (Experiment 1). Forty-one participants, aged 21–31, rated each human-robot face presented for either a brief (50 ms) or long duration (3 s) in terms of attractiveness, eeriness, and humanness (UV indices) in a 7-point Likert scale. We found that brief and long exposures to stimuli generated a similar UV effect. This suggests that the UV effect is accessible at early visual processing. We then examined the effect of exposure duration on the categorisation of visual stimuli in Experiment 2. Thirty-three participants, aged 21–31, categorised faces as either *human* or *robot* in a two-alternative forced choice task. Their response accuracy and variance were recorded. We found that brief stimulus exposures generated significantly higher response variation and errors than the long exposure condition. This indicated that participants were more uncertain in categorising faces in the brief exposure condition due to insufficient time. Further comparisons between Experiment 1 and 2 revealed that the eeriest faces were not the hardest to categorise. Overall, these findings indicate (1) that both the UV effect and categorical uncertainty can be elicited through brief stimulus exposure, but (2) that categorical uncertainty is unlikely to cause the UV effect. These findings provide insights towards the perception of robotic faces and implications for the design of robots, androids, avatars, and artificial intelligence agents.

## 1 Introduction

Fear is a typical response to quasi-humans. Past research has shown that near-human appearances of artificial entities such as dolls [1], clowns [2], zombies, and aliens [3] create feelings of discomfort. More recently, androids, computer generated characters, and avatars have also elicited a similar but distinct kind of eeriness [4]. With the rising popularity of human-like robots and artificial intelligence models [5], engineers and artists face mounting challenges to avoid generating these feelings of eeriness through their creations [3]. Therefore, questions surrounding the discomfort associated with near-human entities – also known as the uncanny valley effect – have become a topic of interest and importance.

### 1.1 The uncanny valley and its hypotheses

**Fig. 1:**
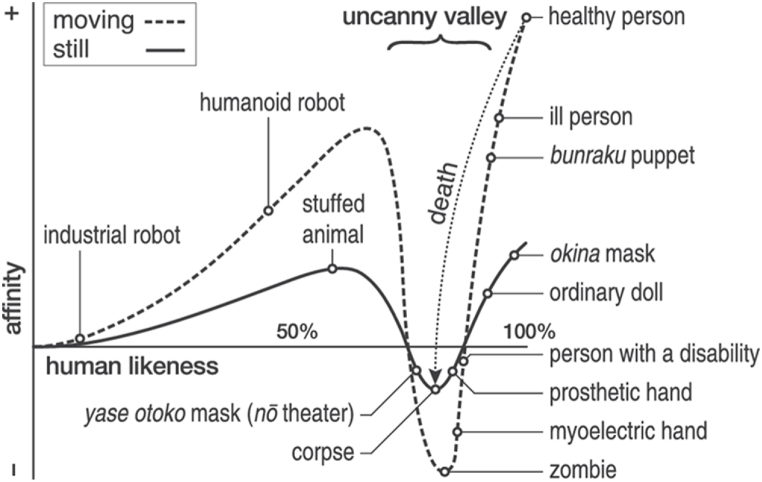
Representation of Mori's UV curve. Adapted from “Creepy cats and strange high houses: Support for configural processing in testing predictions of nine uncanny valley theories” by A. Diel, and K. F. MacDorman (2021), *Journal of Vision,* 21(4), p. 2. Copyright 2021 by the Authors. Published under CC BY 4.0 DEED license.

The concept of uncanny valley (*bukimi no tani* in Japanese) was initially introduced by Japanese roboticist Masahiro Mori in 1970 [6]. The UV proposes that our affinity for representations become stronger as they become more human-like, reaching a peak before a sudden descent into revulsion when these representations are “almost like humans but not quite authentically human” [7]. This nonlinear relation between human-likeness or similarity to humanness (x-axis) and affinity (y-axis) is represented as the UV curve [8,9] (Fig. 1).

Many attempts have been made to verify the UV effect and to explain the UV effect through theories and hypotheses [7,[10], [11], [12]]. For example, the pathogen avoidance hypothesis [13,14] states that uncanny stimuli have imperfections that may signal transmissible diseases, hence triggering avoidance and disgust. The mortality salience hypothesis states that uncanny stimuli may remind observers of their mortality and hence trigger the fear of death. The violation of expectation hypothesis proposes that uncanny feelings are generated by entities creating expectations for a human but then violating these expectations [14] (e.g., mismatches between the visual and tactile quality of prosthetic hands [6] or the combination of a synthetic voice and human appearance in androids [15]). The mind perception hypothesis argues that uncanny feelings are generated by attributing human experience to nonhuman entities, an act that upsets the intuition of the human mind.

**Fig. 2:**
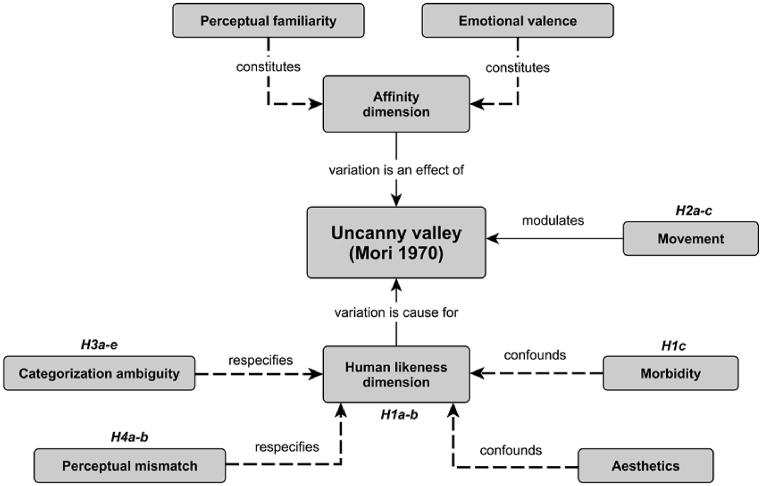
Concept map of various UV constructs and hypotheses derived from Mori (1970/2012). From “A review of empirical evidence on different uncanny valley hypotheses: support for perceptual mismatch as one road to the valley of eeriness” by J. Kätsyri, K. Forger, M. Mäkäräinen, T. Takala (2015), *Frontiers in Psychology*, 6, p. 5. Copyright 2015 by Frontiers Journals. Published under CC BY 4.0 license.

Such theories were previously grouped into two categories: one type of theory considered the UV as an “automatic, stimulus-driven, specialized processing that occurs early in perception”, while the other type of theory considered the UV to be “a broader and more general range of cognitive processing that occurs later” [12,13]. More recent approaches have divided UV theories into more precise subtypes [9,10] (see Diel & MacDorman (2021) for the 9 classes of UV theories and Fig. 2). Among these theories, some researchers have noted that the perceptual mismatch and categorical uncertainty hypotheses have received the most support in the literature [10,16]. These hypotheses state that the UV effect arises when an entity's features belong to different conceptual categories (perceptual mismatch), or when there is conflict over the category membership of an entity (categorical uncertainty) [10,17].

Yet despite efforts to test UV theories, it has been challenging to find a common understanding of UV mechanisms [11]. To address this research gap, we investigate a fundamental aspect of the UV – the visual processing of entities that generate the UV effect.

### 1.2 Levels of visual processing

The hierarchical visual processing framework proposes that visual information processing starts from the primary visual cortex, which processes simple visual features (e.g., orientation and direction) to complex stimuli such as faces [18]. Increased neuronal response latency has been observed to reflect the increasing complexity of visual stimuli. For example, the primary visual cortex processes information as early as around 50 ms to a uniform surface after stimulus onset, whereas the face areas (Fusiform Gyrus) take about 100 ms to process faces.

Behaviourally, face perception can be generated by stimuli presented for as short as 30–38 ms for facial emotions [19] and 100 ms for the judgement of trustworthiness [20,21]. Previous research has shown that participants can judge the orientation of bars and facial emotions of cartoon faces after only a 35 ms presentation of the stimulus [22]. However, it takes about 200 ms of stimulus presentation for participants to judge the emotions of real faces [23]. Furthermore, the perception of facial emotions can be biased by previous visual experience (e.g., an angry face) that were exposed for as brief as 17 ms [24]. In addition to facial emotions and trustworthiness, other facial attributes can be formed in face perception, such as attractiveness, valence, dominance, threat, facial maturity, and femininity-masculinity. These facial attributes are not independent. It has been shown that attractiveness is highly correlated with trustworthiness and valence [25], and that happy faces appear more attractive than neutral faces [26].

These findings therefore raise the question of whether faces can still generate the UV effect when the stimulus presentation time is very short (50 ms). If we prolong the stimulus presentation time to 3 s, could this judgement of the UV effect change? Are there any differences in the UV effect created by faces (measured by a face's attractiveness, eeriness, and perceived humanness) presented for 3 s compared to 50 ms? In the first experiment, we address these questions.

### 1.3 The present study

To address the research questions, the present study investigates whether the UV effect occurs at early or late visual information processing. We manipulated the stimulus presentation duration and examined its effect on our judgement of attractiveness, eeriness, and humanness (the indicators of the UV effect). As previous findings in neuroscience and psychophysical studies illustrated that approximately 20–50 ms is usually needed for simple stimulus presentation, and more than 200 ms is needed for complex visual stimuli, we presented human-robot faces for 50 ms and 3 s to detect any differences in perceived UV effect. If 50 ms is sufficient to generate the UV effect, it may imply that the UV relies more upon automatic fast processes than time-consuming processes.

Recent works have found more support for perceptual mismatch and configural processing theories, perhaps indicating that the UV effect occurs early in visual processing. We test our first hypothesis in Experiment 1.

H1 Brief exposure to human-robot faces generates similar levels of attractiveness, eeriness, and humanness in participants as long exposure.

## 2 Experiment 1

### 2.1 Methods

Prior to the experiment, an *a priori* power analysis was conducted using G*Power (Version 3.1) to calculate the minimum sample size. In anticipation of the nonlinearities of the UV data, we determined that a sample size of *n* = 32 was needed to achieve 0.80 power for detecting a large effect (*f*^*2*^ = 0.35) [27] under multiple linear regression analysis (*α* = 0.05; 1 − *β* = 0.81). We chose to detect a large effect size because most neural mechanisms exhibit a pattern of temporal consistency across people [[28], [29], [30]]. By extension, we expect the UV effect to occur strongly either in the early or late-stage visual processing level across our participants.

#### 2.1.1 Participants

Forty-one participants aged between 21 and 31 were recruited for Experiment 1 at Nanyang Technological University (NTU), Singapore. This was to control possible age effects associated with face perception and recognition [31]. Participants diagnosed with previous or ongoing neurological diseases were also excluded from this study. Participants were openly recruited via social media channels and online advertisements and were remunerated five Singapore dollars in exchange for their involvement.

Recruitment and data collection ran for a week from March 23, 2021 and March 31, 2021, respectively. All participants provided informed consent before the start of the experiment and were debriefed by the co-investigator. In accordance with the main language of instruction of the university, all study components were administered in English. This study has been approved by the Ethics Committee of Psychology Programme, NTU, Singapore under PSY-IRB-2021-002.

#### 2.1.2 Apparatus

This study was conducted at the Visual Perception Lab at NTU. Participants were tested in a dimly lit lab for the experiment with PsychToolbox of Matlab (Version R2020b; the Mathworks, MA, USA) installed in the 21-inch iMac (Apple, CA, USA) with 75 Hz refresh rate.

#### 2.1.3 Visual stimuli – a validated Face corpus

**Fig. 3:**
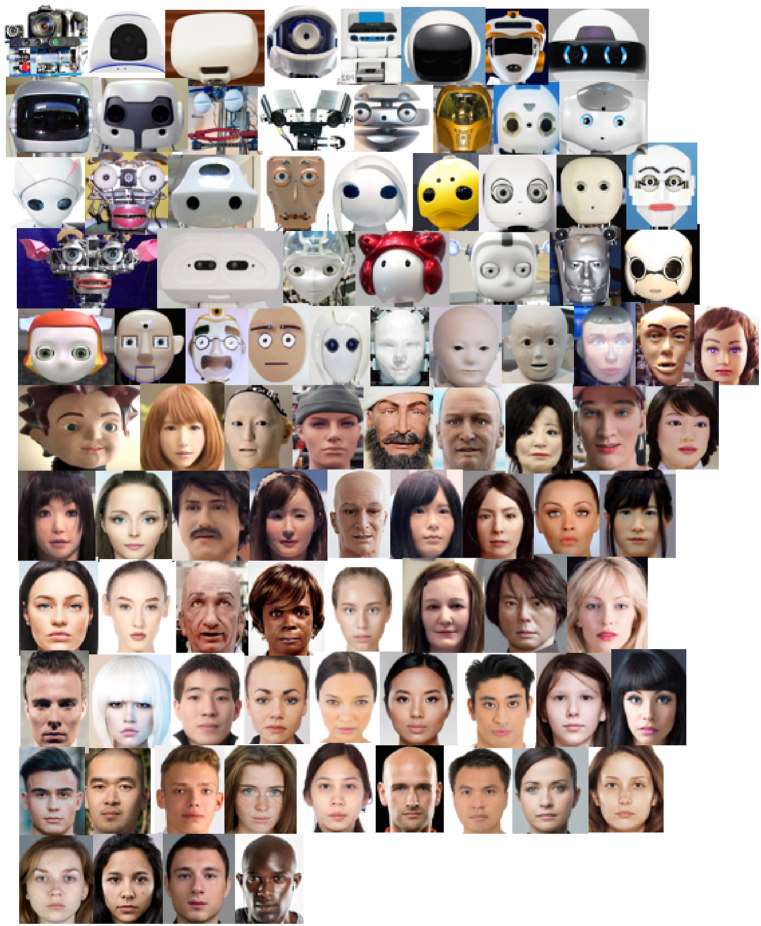
Sample faces extracted from validated face corpus. Adapted from ‘Uncanny but not confusing: Multisite study of perceptual category confusion in the UV’ by Mathur et al. (2020), *Computers in Human Behaviour,* 103, p. 24. Copyright 2019 by Elsevier Ltd. Published under CC BY-NC-ND 4.0 DEED license. *Note.* Faces are arranged from lowest to highest MH score, following Mathur et al. (2020).

The stimuli used in the present study come from a validated face corpus designed to accurately reflect the controlled progression from mechanical to human faces [32]. The face corpus consists of 182 images with 122 robot and 60 human faces and was assembled via a systematic internet search of socially interactive robots and human faces. These faces were selected to obtain a dense spread of mechano-humanness (MH) score on a continuous range from −100 (extremely mechanical) to +100 (extremely human-like). The finalised face corpus was also validated in the original study from Mathur and colleagues [32]. The face corpus is publicly available on OpenScience Framework (https://osf.io/2v6f4/) for by-attribution use. Due to time restrictions, 91 faces were extracted from the validated face corpus for use in the present study. They were obtained by systematically removing every alternate face in ascending order of mechano-humanness (MH) score to preserve the original distribution as best as possible. It should be noted that there is no clear category boundary in the stimulus. Some robot faces may approach the human end-point of the MH spectrum – due to being categorically ambiguous – and hence mix with real human faces in MH order. Therefore, the final test stimuli consisted of 63 robot and 28 human faces (Fig. 3).

#### 2.1.4 Uncanny valley indices

Three UV indices, attractiveness, eeriness, and humanness, developed by Ho and MacDorman were designed to measure the UV effect experienced by our participants. They each constituted several semantic differential scales, such as ugly-beautiful (attractiveness), numbing-freaky (eeriness) and synthetic-real (humanness). When combined to produce an overall score, the scales corresponding to the attractiveness, eeriness, and humanness indices yielded Cronbach's alphas of 0.90, 0.71, and 0.92, respectively [33].

We considered the possibility that our participants might not be able to answer all the items based on first impressions as their exposure to the stimuli may be extremely brief. Thus, we aimed to select as few items as possible and extracted the scales with the highest factor loading from each index to use. These scales correlated the most with their respective UV indices out of all available scales and would provide the best measurement as individual items. They were unattractive-attractive, reassuring-eerie and artificial-natural with a factor loading of 0.87, 0.79, and 0.89. Participants rated the resulting 7-point semantic differential scales.

• How unattractive or attractive is this face? (1 = *unattractive*, 7 = *attractive*)
• How reassuring or eerie is this face? (1 = *reassuring*, 7 = *eerie*)
• How artificial or natural is this face? (1 = *artificial*, 7 = *natural*)

Higher scores denoted greater levels of reported attractiveness, eeriness, and humanness for a face. These items yielded a high Cronbach's alpha of 0.92 for overall UV effect in the present study. We used these indices to measure the UV effect in Experiment 1.

#### 2.1.5 Procedure

Each participant was randomly assigned to the brief (50 ms) or long exposure condition (3 s) by drawing lots before the start of the experiment. When ready, participants were then presented with a human-robot face for either 50 ms or 3 s depending on their assigned test conditions. Immediately after each face was presented, its pixels were scrambled to produce and present a visual noise mask for 1 s. According to the backwards masking paradigm in visual perception research, noise masking is an effective method to reduce visual afterimages and hence limit face processing post-exposure [34,35]. This method allowed us to better limit our participants’ ability to process faces in the brief exposure condition. In both test conditions, the order of the 91 faces and their corresponding masks were fully randomised to minimise order effects.

**Fig. 4:**
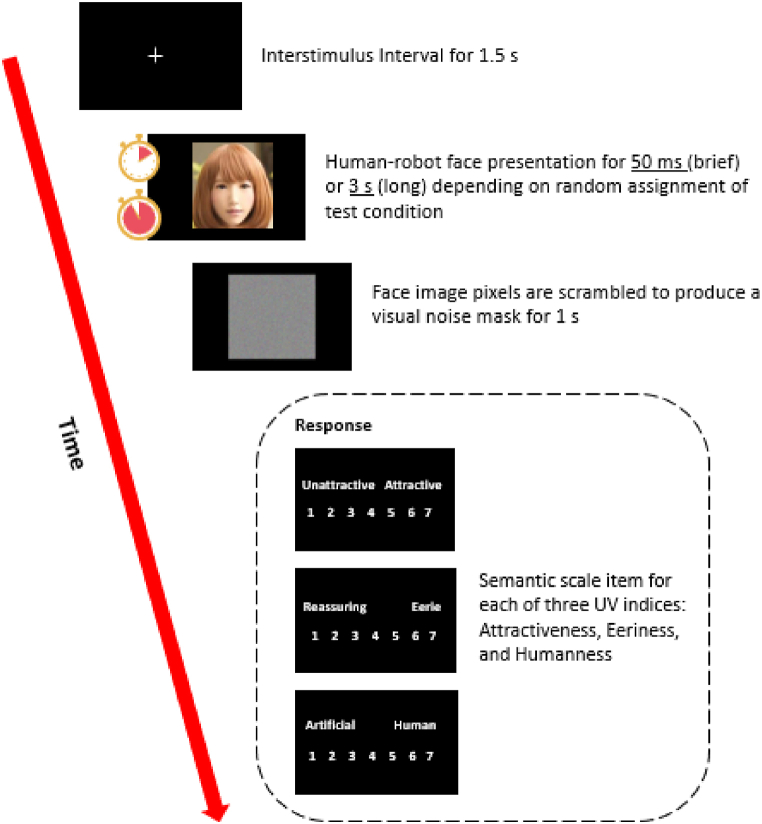
Trial sequence for a human-robot face rating in Experiment 1 (*n* = 41; *S* = 91).

In Experiment 1, participants were then prompted to rate each face on three UV indices – attractiveness, eeriness, and humanness [36]. Each face appeared only once and the order of the questions were held constant throughout the experiment, such that response latency would not reflect the time taken to re-read the questions. An interstimulus interval consisting of a white cross at the centre of the screen then appeared for 1.5 s. These steps, from human-robot face presentation to the interstimulus interval, constituted one trial sequence (Fig. 4). Participants were shown and rated all 91 visual stimuli. The average time taken to complete all trials and sample size of each experiment group is provided in Table 1.

**Table 1:** Average time taken and sample size (Experiment 1).

	Condition	Average time to complete (mins)	Sample size (n)
Experiment 1	Brief	10.70	20
	Long	15.32	21

#### 2.1.6 Statistical analysis

We used statistical software Jamovi (Version 1.6.15) [37] for data analysis. To examine if the duration of exposure affected the UV effect towards human-robot faces (H1), a series of multiple polynomial regressions were conducted. We chose to perform polynomial regressions to accommodate the expected non-linearities of the UV curve against MH score. Each polynomial regression accounted for attractiveness, eeriness, and humanness individually, as these indices were de-correlated in the original scale [36]. Therefore, a preliminary correlation analysis was performed between the main variables of interest to check if the UV indices remained de-correlated after excluding majority of these correlated items (as described in Section 2.1.4).

Using the Jamovi GAMLj module, each UV index was entered as the dependent variable, while MH score and the duration of exposure were entered as continuous and categorical predictors, respectively. To determine the polynomial term, MH score started from the highest degree (i.e., fifth degree in Jamovi) and was reduced stepwise to select the most parsimonious model using the backward elimination method. This was performed by comparing *F*-test scores across the regression models produced [38].

Lastly, inspection of the raw data revealed that one participant of Experiment 1 used the same response for most items. Therefore, this participant's data were attributed to extremity bias and were excluded from further analysis. This left 40 participants' data in Experiment 1 for our polynomial analyses. Our study's data, materials and analysis scripts for both Experiments 1 and 2 are publicly available for by-attribution use (https://osf.io/y3ah8/).

### 2.2 Results of experiment 1

As we had preserved the original distribution of MH scores from the face corpus [38], and that the UV effect observes a vastly different pattern from normal distributions, it is expected that preliminary analysis of the data would violate the normality assumption. This was confirmed by Shapiro-Wilk tests of normality on all three measures of UV – attractiveness (*W*_*brief*_ = 0.81, *p* < 0.001; *W*_*long*_ = 0.83, *p* < 0.001), eeriness (*W*_*brief*_ = 0.95, *p* < 0.001; *W*_*long*_ = 0.97, *p* = 0.049), and humanness (*W*_*brief*_ = 0.91, *p* < 0.001; *W*_*long*_ = 0.97, *p* = 0.026) (Table 2 for detailed statistics).

**Table 2:** Descriptive statistics of main variables by group (Experiment 1).

	Condition	Attractiveness	Eeriness	Humanness
Mean	Brief	2.84	4.70	3.27
	Long	2.99	4.59	3.21
Standard deviation	Brief	1.93	1.20	1.28
	Long	1.90	1.11	1.10
Skewness	Brief	0.46	−0.24	0.31
	Long	0.73	−0.28	0.33
Kurtosis	Brief	−1.47	−1.14	−1.34
	Long	−1.00	−0.56	−0.76
Shapiro-Wilk W	Brief	0.81***	0.95***	0.91***
	Long	0.83***	0.97*	0.97*

*Note.* Participants: *n* = 40. Face Stimuli: *S* = 91.

*p < 0.05, **p < 0.01, ***p < 0.001.

To find the associations between the variables measured, we used nonparametric Kendall's tau for correlation analysis (Table 3) as the data was not normally distributed. Significant associations were found between attractiveness, eeriness, and humanness, which violated the decorrelation of indices in Ho and MacDorman's scale [36]. This poses a limitation to our study that we will discuss in later sections. Significant associations were also found cross-sectionally between MH score and the three UV indices. We observed that eeriness was negatively correlated with attractiveness (*r* = −0.62, *p* < 0.001), which was an expected pattern as they evoke opposing affective states [10,39]. Eeriness was also negatively correlated with humanness (*r* = −0.79, *p* < 0.001), and MH score (*r* = −0.44, *p* < 0.001), which suggested that more human-like faces evoked less feelings of eeriness. However, we cannot interpret this correlation at face value given that positive affect increases non-linearly with the human-likeness dimension. As expected, humanness and MH score were positively correlated (*r* = 0.44, *p* < 0.001) due to the construction of the face corpus used.

**Table 3:** Kendall's tau correlation of main variables (Experiment 1).

	Attractiveness	Eeriness	Humanness	Mechano-Humanness Score
Attractiveness	–			
Eeriness	−0.62***	–		
Humanness	0.58***	−0.79***	–	
Mechano-Humanness Score	0.73***	−0.45***	0.44***	–

*Note*. Participants: *n* = 40. Face Stimuli: *S* = 91.

*p < 0.05, **p < 0.01, ***p < 0.001.

#### 2.2.1 Does duration of exposure affect the UV effect?

To quantitatively model the relationship between MH score, exposure duration and each of the UV indices, we conducted a series of polynomial regressions using the backward elimination method. We found that the MH score was significant up to the quadratic term for all three UV indices when compared to the models of cubic terms (*F*_*att*_ (1, 178) = 183.93, *p* < 0.001; *F*_*eer*_(1, 178) = 105.88, *p* < 0.001; *F*_*hum*_(1, 178) = 41.43, *p* < 0.001). Thus, our final models included MH score of up to the second-degree polynomial term.

**Fig. 5:**
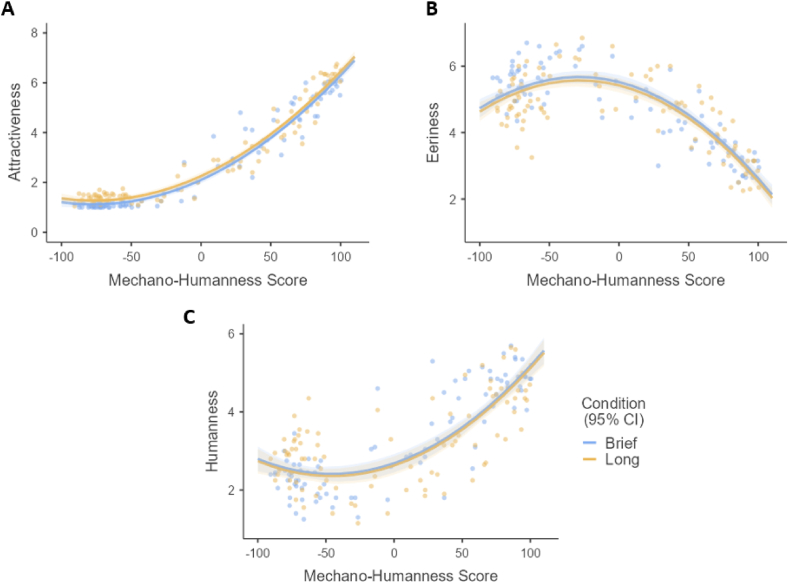
Second-order multiple polynomial regression plots regressing UV Indices onto exposure condition. (A) Attractiveness on MH Score and exposure condition. (B) Eeriness on MH Score and exposure condition. (C) Humanness on MH Score and exposure condition.

The plots for each multiple polynomial regression are compiled in Fig. 5. Their estimated regression coefficients and their confidence intervals are in the Supplementary Material (Supplementary Tables 1–3).

All the models showed nonlinear relations between the UV indices (Attractiveness, Eeriness, and Humanness) and the MH Score. The first and second order of MH score were significant in all models (Supplementary Tables 1–3). However, the duration of exposure (Condition) was not significant in the Eeriness and Humanness models, which suggested that the brief and long exposure conditions generally had similar effects on the UV indices. The data thus supports H1. The effect of exposure duration on Attractiveness is also explained below.

To predict attractiveness from MH score and exposure duration, the first multiple polynomial regression revealed the equation: Attractiveness = 2.10 + 0.08 (Condition) + 0.90 (MH Score) + 0.39 (MH Score)^2^. Our model was significant (*F*(3, 178) = 1058.93, *p* < 0.001) with an adjusted R^2^ of 0.95. Omnibus tests showed significant main effects on attractiveness by both first-degree and second-degree MH terms (*F*_*MH*_^*1*^(1, 178) = 2585.50, *p* < 0.001, *η*_*p*_^*2*^ = 0.94; *F*_*MH*_^*2*^(1, 178) = 183.93, *p* < 0.001, *η*_*p*_^*2*^ = 0.51) and by exposure duration (*F*(1, 178) = 1.03, *p* = 0.03, *η*_*p*_^*2*^ = 0.03). This indicated that mean attractiveness significantly differed between exposure conditions (Fig. 5A), and data on the attractiveness index did not support H1. However, considering that participants rated faces as slightly more attractive in the long exposure condition (*M* = 2.99) compared to the brief exposure condition (*M* = 2.84), this indicates that processing attractiveness at a higher level did not result in a meaningful increase in UV effect.

The second model predicting eeriness from MH score and duration of exposure generated the equation: Eeriness = 5.47 - 0.09 (Condition) - 0.62 (MH Score) - 0.72 (MH Score)^2^. Our model was significant (*F*(3, 178) = 132.86, *p* < 0.001) with an adjusted R^2^ of 0.68. Omnibus tests showed significant main effects on eeriness by both first-degree and second-degree MH terms (*F*_*MH*_^*1*^(1, 178) = 204.39, *p* < 0.001, *η*_*p*_^*2*^ = 0.54; *F*_*MH*_^*2*^(1, 178) = 105.88, *p* < 0.001, *η*_*p*_^*2*^ = 0.37). However, non-significant effects were observed by the duration of exposure (*F*(1, 178) = 1.10, *p* = 0.30, *η*_*p*_^*2*^ = 0.006). This indicated that mean eeriness did not significantly differ between exposure conditions (Fig. 5B). Thus, data on the eeriness index did support H1.

The third model predicting humanness from MH score and duration of exposure resulted in the equation: Humanness = 2.69 - 0.05 (Condition) + 0.67 (MH Score) + 0.49 (MH Score)^2^. Our model is significant (*F*(3, 178) = 133.95, *p* < 0.001) with an adjusted R^2^ of 0.62. As with eeriness, omnibus tests showed significant main effects on humanness by both first-degree and second-degree MH terms (*F*_*MH*_^*1*^(1, 178) = 205.01, *p* < 0.001, *η*_*p*_^*2*^ = 0.54; *F*_*MH*_^*2*^(1, 178) = 41.43, *p* < 0.001, *η*_*p*_^*2*^ = 0.19) but non-significant effects by duration of exposure (*F*(1, 178) = 0.27, *p* = 0.60, *η*_*p*_^*2*^ = 0.002). This indicated that mean humanness did not significantly differ between exposure conditions (Fig. 5C). As with the eeriness index, data on the humanness index also supports H1.

Besides the UV indices, participants' response latencies were also likely affected by MH score and exposure duration. However, we excluded these findings as participants’ response latencies may have been influenced by other factors in the human-robot rating task, such as emotional arousal caused by the stimuli [40,41]. We noted that past psychophysical experiments studying visual perception often used a two-alternative forced choice (2-AFC) paradigm instead (e.g., to judge facial emotions [23]). To derive more information into the UV effect, we used a 2-AFC task wherein participants categorised each visual stimulus as *human* or *robot* in Experiment 2.

## 3 Experiment 2

Brief exposure to facial stimuli often makes recognition and categorisation tasks more difficult. The ability to categorise varies with the task involved. For example, gender and familiarity categorisations can occur with 100 ms of stimulus exposure, but higher-order categorisations typically need stimulus exposures of 300 ms and above [42,43]. Therefore, we question whether varying stimulus exposures will impact the accessibility, and hence certainty of anthropomorphic face categorisation. If so, is this (un)certainty related to the UV effect observed in Experiment 1?

Categorical uncertainty refers to a feeling of confusion when an entity's ambiguous appearance broaches the boundary between two discrete categories (e.g., human vs. nonhuman) [16,44]. In the UV, the categorical uncertainty hypothesis states that the cognitive barriers from resolving unclear category boundaries for between-human entities create feelings of unease and aversion [[45], [46], [47]]. Previous research found that categorical conflict coincided with the lowest likeability ratings and mind attribution of [17] anthropomorphic faces. However, more recent studies found that manipulating categorical uncertainty had either no negative effect, or that the eeriest stimuli were not necessarily categorically ambiguous [32,48]. Researchers acknowledge the existence of categorical uncertainty in the UV, but robust evidence has yet to show that it causes the UV effect [10].

To substantiate such evidence, we test whether categorical uncertainty occurs with the UV effect. Previous studies in visual perception suggest that brief exposures to oriented bars can activate the global configuration of stimuli [49], contributing to the holistic perception of a face [22]. For brief visual stimuli exposures, we hypothesise that participants may not be able to observe the stimuli's features in detail, and thus overlook visual features and categorise stimuli using holistic processing with high uncertainty. For longer visual stimuli exposures, participants have more time to examine the details of visual stimuli, and are likely to categorise stimuli using both local and holistic processing with less uncertainty. We arrived at this hypothesis as UV literature often discusses categorical uncertainty in the UV as a ‘cognitive’ or ‘late-stage’ process. Therefore, the following hypotheses are proposed:

H2a Brief stimulus exposure (50 ms) increases categorical uncertainty in participants' responses (i.e., higher response variance and lower response accuracy) compared to long exposure (3 s).

H2b If categorisation uncertainty explains the observed UV effect, we expect the eeriest stimuli to be associated with the largest response uncertainty (i.e., highest response variance and lowest response accuracy).

### 3.1 Methods

#### 3.1.1 Participants, Apparatus, and visual stimuli

Using the same *a priori* power analysis (*n* = 32; *α* = 0.05; 1−*β* = 0.81) to detect a large effect size (*f*^*2*^ = 0.35), 33 participants were recruited for Experiment 2 at NTU. Participants were 42.4% male and 57.6% female, ranged from ages 21 to 33 with a mean age of 23.67 (SD = 2.85). Of the participants, 87.9%, 0.09% and 0.03% were of Chinese, Indian, and Sinhalese ethnicities, respectively. All participants had attained a post-secondary qualification or above. Recruitment and data collection ran for about a week from September 25, 2023 and September 26, 2023, respectively. As with Experiment 1, the study was conducted in a quiet dimly lit lab at NTU (Visual Cognitive Neuroscience Lab) with the same set of visual stimuli and procedures.

**Fig. 6:**
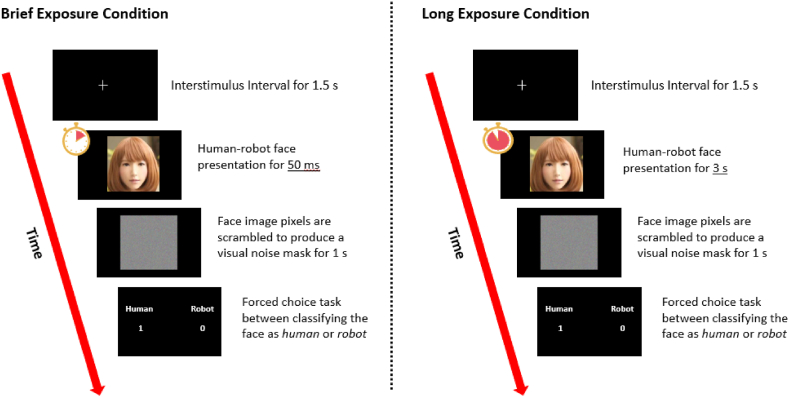
Trial sequence for a human-robot face for Experiment 2 (*n* = 33; *S* = 91). *Note.* Each participant completed human-robot ratings of 91 visual stimuli presented in random order in the brief exposure condition (left). After a 5-min break, they were tested under the long exposure condition (right) with the same visual stimuli in random order.

Unlike Experiment 1, Experiment 2 used a within-subjects design and tasked participants to categorise each face as either *human* or *robot* (Fig. 6). Participants were exposed to all 91 visual stimuli (same as those in Experiment 1) for 50 ms (brief exposure condition) before repeating each stimulus for 3 s (long exposure condition) in random order. Each stimulus was preceded by an interstimulus interval for 1.5 s, and was immediately followed by a visual mask for 1 s and then the human-robot categorisation in a 2-AFC task. Participants were also given a 5-min break between conditions to minimise the effect of memory associated with the visual stimuli. Participants (*n* = 33) took 11.06 min on average to complete Experiment 2.

Prior to Experiment 2, we also recruited 37 participants from NTU to a preliminary run using a between-subject design. They were randomly assigned to either the brief or long exposure condition and completed the human-robot categorisation task once per face. The between-subjects data can be found in the Supplementary Material (descriptive data in Supplementary Tables 4–5; main analyses in Supplementary Fig. 1 and Supplementary Tables 6–7). However, as each participant's processing capacities are unique and can serve as their own control, we had collected the within-subject data for the main analysis.

#### 3.1.2 Measure of categorical uncertainty

Categorical uncertainty is often tested by evaluating affective or physiological indicators such as a participant's response latency when categorising stimuli. Other measurements, such as response accuracy and response variance, may also indicate categorical uncertainty. Response accuracy measures the degree to which the responses are correct. In this experiment, it refers to the percentage of responses consistent with the actual human/robot category of the visual stimuli, and was calculated by dividing the number of correct responses by the total number of responses to each stimulus. Response variance measures the degree to which individual responses deviate from one another, and refers to the spread of human or robot responses to each stimulus. Here, a response categorising the visual stimulus as human was assigned the value ‘1’ and robot ‘0’. The response variance for each stimulus was calculated using the formula below:

$$\sigma^{2}=\frac{1}{N}\sum_{i=1}^{N}{\left(x_{i}-\mu \right)}^{2}$$

Where:

$\sigma^{2}$ is variance;

N is the number of responses to a visual stimulus;

$x_{i}$ is the individual response (‘1’ for human and ‘0’ for robot);

μ is the mean of responses.

Response latency was measured in seconds after the offset of the visual stimulus. It was not highlighted as a measure of categorical uncertainty as participants may naturally take a longer time to respond in the long stimulus exposure condition, since longer durations are required to process visual scenes beyond their gist [50]. Thus, we focus on the other two measures: response variance and response accuracy in the following sections.

#### 3.1.3 Statistical analysis

After preliminary and correlation analyses like in Experiment 1, polynomial regressions were performed to examine if the duration of exposure affected categorical uncertainty across conditions (H2a). We performed polynomial regressions on response variance and response accuracy to plot their relationship with faces on the MH spectrum. Despite the option of a multivariate analysis, we performed the polynomial regressions for response variance and response accuracy individually to maintain consistency with the de-correlation of UV indices in Experiment 1. One participant in Experiment 2 was excluded for reporting a neurodevelopmental condition. This left 32 participants’ data from Experiment 2 for further analysis.

### 3.2 Results of experiment 2

Like Experiment 1, preliminary analysis of Experiment 2 data showed that the data violated the normality assumption – Shapiro-Wilk tests of normality on response variance (*W*_*brief*_ = 0.78, *p* < 0.001; *W*_*long*_ = 0.70, *p* < 0.001) and response accuracy (*W*_*brief*_ = 0.79, *p* < 0.001; *W*_*long*_ = 0.61, *p* < 0.001) were significant across both test conditions. The preliminary analysis and descriptive statistics are reported in Table 4. Although we excluded response latency as a measurement in the main analysis, we included it below for descriptive purposes. However, the interpretation of response latency must be taken with caution for reasons mentioned in Section 3.1.2.

**Table 4:** Descriptive statistics of main variables by group (Experiment 2).

	Condition	Response Variance	Response Accuracy	Response Latency (seconds)^a^
Mean	Brief	0.09	0.85	0.46
	Long	0.06	0.90	0.49
Standard deviation	Brief	0.10	0.18	0.10
	Long	0.09	0.18	0.08
Skewness	Brief	0.44	−1.15	1.12
	Long	1.25	−2.19	1.29
Kurtosis	Brief	−1.56	0.42	1.43
	Long	−0.02	4.07	2.40
Shapiro-Wilk W	Brief	0.78***	0.79***	0.92***
	Long	0.70***	0.61***	0.91***

*Note*. Participants: *n* = 32. Face Stimuli: *S* = 91.

*p < 0.05, **p < 0.01, ***p < 0.001.

a Response latency was measured in seconds after the offset of the stimulus.

Nonparametric Kendall's tau was used for the correlation analysis (Table 5). We found significant associations between response variance, response accuracy, response latency, and MH score: response variance and response accuracy were negatively correlated (*r* = −0.98, *p* < 0.001); both response latency and MH score were positively correlated with response variance (*r*_*latency*_ = 0.25, *p* < 0.001; *r*_*MH*_ = 0.46, *p* < 0.001, respectively) but negatively correlated with response accuracy (*r*_*latency*_ = −0.25, *p* < 0.001; *r*_*MH*_ = −0.46, *p* < 0.001, respectively). Further regression analyses were performed to visualise and evaluate the extent of these dependencies as with Experiment 1.

**Table 5:** Kendall's tau correlation of main variables (Experiment 2).

		Response Variance	Response Accuracy	Response Latency	MH Score
Response Variance	Kendall's tau B	–			
Response Accuracy	Kendall's tau B	−0.98***	–		
Response Latency	Kendall's tau B	0.25***	−0.25***	–	
MH Score	Kendall's tau B	0.46***	−0.46***	0.05	–

*Note*. Participants: *n* = 32. Face Stimuli: *S* = 91.

*p < 0.05, **p < 0.01, ***p < 0.001.

#### 3.2.1 Does duration of exposure affect categorical uncertainty?

**Fig. 7:**
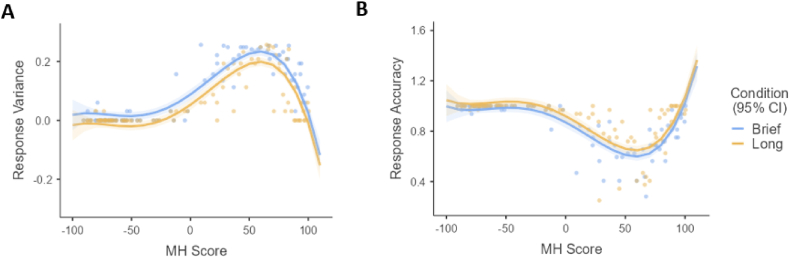
Fourth-order polynomial regressions of categorical uncertainty measures onto MH Score and exposure condition. (A) Response Variance on MH Score and exposure condition. (B) Response Accuracy on MH Score and exposure condition.

Using the data from Experiment 2, we test H2a – participants in the brief exposure condition will report higher response variance and lower response accuracy compared to those in the long exposure condition. Response variance and accuracy scores were entered as the dependent variables while keeping the MH score and exposure duration as independent variables. Using the same backward elimination methods in Experiment 1, our findings showed that MH score was significant up to the quartic term for response variance (*F*_*variance*_(1,175) = 26.73, *p* < 0.001) and response accuracy (*F*_*accuracy*_(1,175) = 21.54, *p* < 0.001) when comparing against the quintic term. Thus, our final models included MH score up to the fourth-degree polynomial term. The plot for this regression is illustrated in Fig. 7, and its estimates and 95% C.I.s can be found in the Supplementary Material (Supplementary Tables 8–9).

The resulting model predicts response variance from exposure duration (Condition) and MH score by the equation: Response variance = 0.07 – 0.37 (Condition) + 1.99 (MH Score) + 0.93 (MH Score)^2^ – 0.88 (MH Score)^3^ – 0.56 (MH Score)^4^. Our model is significant (*F*(5, 176) = 129.49, *p* < 0.001) with an adjusted R^2^ of 0.78. Omnibus tests showed a significant main effect on response variance by first- to fourth-degree MH terms (*F*_*MH*_^*1*^(1, 176) = 298.39, *p* < 0.001, *η*_*p*_^*2*^ = 0.63; *F*_*MH*_^*2*^(1, 176) = 19.45, *p* < 0.001, *η*_*p*_^*2*^ = 0.10; *F*_*MH*_^*3*^(1, 176) = 104.99, *p* < 0.001, *η*_*p*_^*2*^ = 0.37; *F*_*MH*_^*4*^(1, 176) = 26.79, *p* < 0.001, *η*_*p*_^*2*^ = 0.13) and by exposure duration (*F*(1, 176) = 27.66, *p* < 0.001, *η*_*p*_^*2*^ = 0.13). This indicated that mean response variance differed significantly across exposure conditions (Fig. 7A). Specifically, participants’ response variance of the brief condition (*M* = 0.09) was greater than that of the long condition (*M* = 0.06) with a significant effect. Hence, the data on response variance supports H2a.

Following this, the model predicting response accuracy from MH score and duration of exposure resulted in the equation: Response accuracy = 0.89 + 0.27 (Condition) – 1.86 (MH Score) – 1.07 (MH Score)^2^ + 0.86 (MH Score)^3^ + 0.64 (MH Score)^4^. Our model was significant (*F*(5, 176) = 65.40, *p* < 0.001) with an adjusted R^2^ of 0.64. Omnibus tests showed a significant main effect on eeriness by both first- to fourth-degree MH terms (*F*_*MH*_^*1*^(1, 176) = 159.41, *p* < 0.001, *η*_*p*_^*2*^ = 0.48; *F*_*MH*_^*2*^(1, 176) = 15.43, *p* < 0.001, *η*_*p*_^*2*^ = 0.08; *F*_*MH*_^*3*^(1, 176) = 60.47, *p* = <0.001, *η*_*p*_^*2*^ = 0.26; *F*_*MH*_^*4*^(1, 176) = 21.09, *p* = <0.001, *η*_*p*_^*2*^ = 0.11) and by exposure duration (*F*(1, 176) = 9.10, *p* = 0.003, *η*_*p*_^*2*^ = 0.05). This indicated that the mean response accuracy differed significantly across exposure conditions (Fig. 7B). Similar to response variance, response accuracy of the brief condition (*M* *=* 0.85) was slightly lower than that of the long condition (*M* *=* 0.90), indicating that the data on response accuracy also supports H2a.

## 4 Comparisons of UV effect and categorical uncertainty

**Fig. 8:**
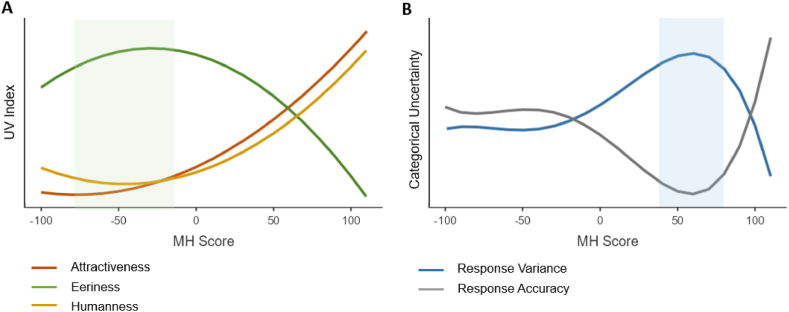
Combined Plots of (A) UV indices and (B) categorical uncertainty on the MH spectrum. *Note.* The areas highlighted in green and blue represent the approximate locations of the maximum/minimum inflection points, respectively. (For interpretation of the references to colour in this figure legend, the reader is referred to the Web version of this article.)

Though we observed both categorical uncertainty and UV effect with brief stimulus exposures, we have yet to observe whether they vary together across the human-likeness dimension (i.e., MH spectrum). We hypothesised that if categorical uncertainty theory is true, the eeriest stimuli should be associated with the greatest categorical uncertainty (H2b). We thus compared the trends between UV indices and categorical uncertainty side-by-side (Fig. 8).

The maximum/minimum inflection points for attractiveness, eeriness, and humanness appear around −50 MH (Fig. 8A), showing that the greatest UV effect occurred nearer to the robot end-point. However, these points appear around +50 to +60 MH for the highest response variance and lowest response accuracy (Fig. 8B), showing that the greatest categorical uncertainty occurred nearer to the human end-point. Together, they show that the eeriest stimuli were not the hardest to categorise by participants. Hence, these comparisons did not support H2b.

## 5 Discussion

### 5.1 Summary of findings

Through manipulating the stimulus exposure duration, we examined its effect on the judgement of the UV. In our first experiment, we asked participants to rate the attractiveness, eeriness, and humanness (UV indices) of visual stimuli presented for either 50 ms or 3 s using a 7-point sematic differential scale. We found that brief and long exposures to the visual stimuli generated similar UV ratings. This effect was captured by nonlinear regression models predicting the strength of UV indices from the human likeness score of the visual stimuli (MH score). Our findings support that the UV effect is elicited by stimulus exposures as brief as 50 ms, implying that the UV is processed at an early and automatic level.

To examine whether stimulus exposure durations affected stimulus categorisation, we conducted the second experiment by asking participants to categorise the visual stimulus (*human* or *robot*) under different exposure durations. We found that brief exposures generated greater errors in response accuracy and response variance compared to long exposures. This showed that fast visual processing was sufficient to elicit categorical uncertainty. However, the observation that categorical uncertainty resolves slightly in long condition may suggest that there was insufficient time for accurate categorisation in the brief condition to begin with.

Lastly, we compared trends between UV indices (Experiment 1) and categorical uncertainty levels (Experiment 2). We found that the UV effect was stronger for faces lower on the human-likeness dimension which did not create much categorical uncertainty. This finding is consistent with the finding from the authors of the face corpus, who found that UV effect occurred at the MH -23.6 range but the category boundary was at the MH +42.5 range [32]. Our study also found the greatest eeriness and categorical uncertainty at approximately MH -25 and MH +50, respectively (Fig. 8).

Results from our first experiment indicate that early visual processing is sufficient to elicit the UV effect. This finding is consistent with the rising empirical evidence for UV theories based on early perceptual cues [9,10,51]. However, brief exposures do not stop perceptual cues being processed further along the cortical hierarchy, as people continue to scrutinise details and interpret the visual stimulus in memory. It might also be that increased visual information may be unnecessary for the UV effect, but enables a more nuanced evaluation available with the high-level processing of secondary sexual characteristics, facial expressions or emotions tied to attractiveness stereotypes. Therefore, brief exposures do not stop the UV effect from being processed at higher levels that may assign cognitive or later-stage processing [13].

The findings from Experiment 2 demonstrated that brief exposures to a visual stimulus likely impaired participants’ ability to categorise human-robot faces. These brief exposures provided limited visual information compared to the long exposures. This implies that though categorical uncertainty is present with brief visual processing, it is associated with an information deficit, so categorical uncertainty associated with visual details may require more time to detect. Regardless, post-hoc comparisons showed that the eeriest stimulus was not categorically ambiguous. They propose that an early visual processing is sufficient to elicit UV and it is unlikely due to categorical uncertainty. This finding is consistent with previous studies that identified the existence of categorical uncertainty in the UV, but also disproved that it specifically causes the UV effect.

### 5.2 Theoretical implications

#### 5.2.1 Etiology of the UV effect

Current researchers have noted that there is a general bias towards measuring cognitive responses over early perceptual processes due to easier manipulation and methodological limitations [12,52]. Yet, our discovery of a low visual processing requirement reinforces the growing emphasis on early perceptual cues as a significant factor to the UV effect in recent works. Our study prompts an important re-examination of the theoretical underpinnings of UV mechanisms wherein stimulus-driven, baseline face-related processes should be given more credit for their etiological contributions to the UV effect.

We also argue that our findings provide indirect evidence for UV theories that rely on early and obvious perceptual cues. For example, theories that rely upon *configural processing* suggest that the UV effect is elicited through deviations in highly specific and familiar stimuli. Such deviations are attributed to invariant arrangements of facial features, which is in turn subject to rapid detection and differentiation of the visual processing system [53]. Theories that rely upon *perceptual mismatches* also purport that the UV effect arises from inconsistencies between the realism levels of specific sensory cues, which is observable through the detection of disproportionate facial features [13,54]. Finally, some theories also discuss *atypicalities* in a broader sense where the UV effect is elicited by an exemplar that deviates strongly from its category prototype regardless of category membership. This is evident from the obvious mismatches in head-body category membership and skin colouration manipulation [55]. We argue that the perceptual cues above are strongly linked to contrast, spatial frequency, colour, and orientation perception [18,56]. The growing evidence supporting this class of theories [9] aligns well with the idea that the UV effect is accessible with minimal visual input.

#### 5.2.2 The relationship between categorical uncertainty and the UV effect

Our study adds to the understanding of categorical uncertainty in the UV knowing that 1) both UV and categorical uncertainty are accessible at early visual processing, but 2) the eeriest stimuli were not the most categorically ambiguous. Our findings support that categorical uncertainty is a natural consequence of uncanny faces but it does not directly explain the UV effect [32,38,48]. Furthermore, superordinate-level categorisations (i.e., *human* versus *robot*) with minimal and added amount of information resulted in similar categorical uncertainty trends across the human-likeness dimension (see Fig. 7). This shows that apart from an information deficit, some categorical uncertainty still occurs with cognitive representations of human-likeness, as longer processing helps participants to detect and scrutinise visual details.

#### 5.2.3 Reviewing the taxonomy of UV theories

Our study lastly gestures towards the importance of a shared taxonomical understanding of UV theories. Given there is no unanimous framework for classifying UV theories, several theories have been noted to have overlapping mechanisms and constructs of interest, begging the question of where these theories had diverged. For example, researchers have noted that the perceptual discrimination hypothesis could be interpreted as the categorical perception hypothesis, depending on category knowledge and the conceptualisation of human-likeness dimension [10]. Likewise, theories with similar mechanisms may be labelled differently depending on their interpretation of which pathways are more important to the UV effect. As our findings assert early-face related processes, further support for the early-perceptual domain could bias the interpretation of complex mechanisms in their favour and influence the formation of hypotheses and concepts surrounding the UV and the presence of human-end points [16].

### 5.3 Practical implications

Beyond improving our understanding of the UV effect, the current study can help to inform the creation of human replicas in the design of virtual worlds or artificial intelligence. As the UV effect appears to be strongly related to early-face related processes, good design principles surrounding robots or avatars can consider avoiding uncanniness through early perceptual cues. For example, good design principles can prioritise exemplary features and avoid perceptual mismatches in the form of both atypicality (e.g., human head on robot body) and realism inconsistency (e.g., robot eyes on human skin). They can keep the arrangement and orientation of facial features as similar as possible to the average human face (e.g., upright faces, proportionate features). Additionally, if the present technology is unable to produce a convincing humanlike appearance, our study implies that foregoing humanlike robots in favour of semi-anthropomorphic robots may ultimately be more acceptable to society and self [57]. This will likely help to reduce the perceived “creepiness” of human-like robots or artificial intelligence models [58].

### 5.4 Limitations and future directions

One major limitation of the present study is that the UV indices plotted against MH score were not fully characteristic of Mori's UV curve (Fig. 1). It should be noted that the quadratic term is not sufficient to visualise the UV, but our method is consistent with that of the original papers and prioritises model parsimony first and foremost. The result was one inflexion point that appears around MH -50 across all models. We expect that this deviation resulted from using 91 out of the 182 faces in Mathur and colleagues (2020)'s face corpus, whereby less densely distributed data might have affected the shape of our polynomial functions. Although the shape of the UV curve is not critical to the investigation of visual processing in the current study, we recommend future studies to use both the complete face corpus and a larger sample size to attain a more precise UV function. This could unveil new patterns in data and comparisons with UV functions attained by other researchers.

Additionally, the de-correlation of UV indices may be due to reducing Ho and MacDorman's (2010) UV indices [44] – where attractiveness, eeriness, and humanness were originally measured by five, eight, and six items, respectively. However, our experiment design is still valid with the fewest items possible, as previously discussed. Future research is encouraged to use all items as suggested by Ho and MacDorman to measure the UV effect if possible [44,47].

Lastly, our study's design is unsuitable for testing theories such as perceptual mismatch theories that rely on early perceptual cues (despite providing indirect support). This is because we used naturalistic images that varied in human-likeness, whereas feature manipulation is required to test these theories. For example, perceptual mismatch theories were tested using 3D computer generated models to adjust the realism of facial features [48,51]. Configural processing theories were tested using Thatcher images [9] or images with disproportionately large eyes [59]. Atypicality theories were tested using morphed sequences as deviations from categorical prototypes [60]. We find that naturalistic images are instead used to test cognitive processes such as mind attribution [61]. Future studies can perform feature manipulation on Mathur et al.’s face corpus with the added benefit of existing human-likeness scores [32].

## 6 Conclusion

We found that both the UV effect and categorical uncertainty occurred at the early visual processing level. Categorical uncertainty was not a qualifying explanation for the UV effect and accurate categorisation of visual details likely takes longer processing. Our results provide indirect support for UV theories that rely on early perceptual mechanisms given that brief visual exposure is sufficient to generate the UV effect. However, the processing of the UV effect further up the cortical hierarchy remains to be answered in future studies. The findings of our study provide insights into the theories for robotic face perception and implications for design of robots, androids, avatars, and artificial intelligence agents.

## Funding

We wish to acknowledge the funding support for this project from 10.13039/501100001475Nanyang Technological University, Singapore under the Undergraduate Research Experience on CAmpus (URECA) programme (YJ), School of Social Sciences, 10.13039/501100001475NTU and AISG (AISG2-RP-2020-019) (HX).

## Data availability statement

The raw data supporting the conclusions of this article will be made available on Open Science Framework: https://osf.io/y3ah8/. Further clarifications on the data will be made upon reasonable request.

## CRediT authorship contribution statement

**Jodie Yam:** Writing – review & editing, Writing – original draft, Visualization, Validation, Software, Resources, Project administration, Methodology, Investigation, Formal analysis, Data curation, Conceptualization. **Tingchen Gong:** Writing – review & editing, Validation, Project administration, Investigation, Data curation. **Hong Xu:** Writing – review & editing, Visualization, Validation, Supervision, Resources, Project administration, Methodology, Investigation, Funding acquisition, Conceptualization.

## Declaration of competing interest

The authors declare that they have no known competing financial interests or personal relationships that could have appeared to influence the work reported in this paper.
