# Supplementary material for: A stimulus exposure of 50 ms elicits the uncanny valley effect
Source: Heliyon. 2024 Mar 12;10(6):e27977. doi: 10.1016/j.heliyon.2024.e27977 (PMC10963319; doi:10.1016/j.heliyon.2024.e27977)
Supplement: Multimedia component 1 [file mmc1.docx]

Supplementary Material


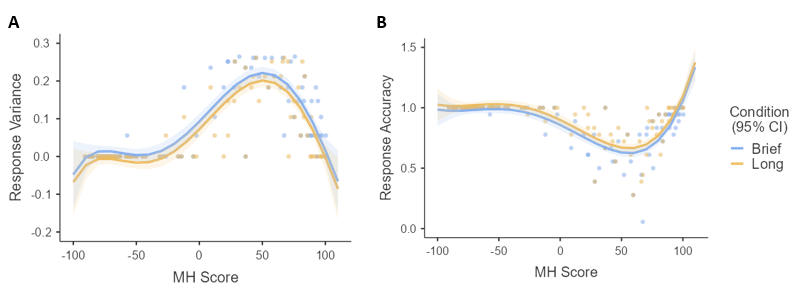


**Supplementary Figure 1.** Fifth- and fourth-order polynomial regressions of categorical uncertainty measures onto MH Score and exposure condition (between-subjects). (A) Response Variance on MH Score and exposure condition. (B) Response Accuracy on MH Score and exposure condition.

# Supplementary Table 1. Regression estimates for polynomial function of Attractiveness on MH Score and Exposure Condition.

| \| Polynomial Model Coefficients: Attractiveness \| \| \| \| \| \| \| \| \| \| \| --- \| --- \| --- \| --- \| --- \| --- \| --- \| --- \| --- \| --- \| \|  \| \| \| \| **95% Confidence Interval** \| \|  \| \| \| \| \| **Names** \| **Effect** \| **Estimate** \| **SE** \| **Lower** \| **Upper** \| **β** \| ***df*** \| ***t*** \| ***p*** \| \| Intercept \| Intercept \| 2.18 \| 0.06 \| 2.05 \| 2.30 \| 0.00 \| 178 \| 34.15 \| < .001 \| \| Condition \| Long - Brief \| 0.15 \| 0.07 \| 0.02 \| 0.28 \| 0.08 \| 178 \| 2.29 \| 0.023 \| \| MH Score \| MH Score \| 0.03 \| 0.00 \| 0.02 \| 0.03 \| 0.90 \| 178 \| 50.85 \| < .001 \| \| MH Score² \| MH Score² \| 0.00 \| 0.00 \| 0.00 \| 0.00 \| 0.39 \| 178 \| 13.56 \| < .001 \| |
| --- | --- | --- | --- | --- | --- | --- | --- | --- | --- | --- | --- | --- | --- | --- | --- | --- | --- | --- | --- | --- | --- | --- | --- | --- | --- | --- | --- | --- | --- | --- | --- | --- | --- | --- | --- | --- | --- | --- | --- | --- | --- | --- | --- | --- | --- | --- | --- | --- | --- | --- | --- | --- | --- | --- | --- | --- | --- | --- | --- | --- | --- | --- | --- | --- | --- | --- | --- | --- | --- | --- |

# Supplementary Table 2. Regression estimates for polynomial function of Eeriness on MH Score and Exposure Condition.

| Polynomial Model Coefficients: Eeriness | | | | | | | | | | | | | | | | | | | |
| --- | --- | --- | --- | --- | --- | --- | --- | --- | --- | --- | --- | --- | --- | --- | --- | --- | --- | --- | --- |
|  | | | | | | | | **95% Confidence Interval** | | | |  | | | | | | | |
| **Names** | | **Effect** | | **Estimate** | | **SE** | | **Lower** | | **Upper** | | **β** | | ***df*** | | ***t*** | | ***p*** | |
| Intercept |  | Intercept |  | 5.47 |  | 0.09 |  | 5.29 |  | 5.66 |  | 0.00 |  | 178 |  | 58.29 |  | < .001 |  |
| Condition |  | Long - Brief |  | -0.10 |  | 0.10 |  | -0.29 |  | 0.09 |  | -0.09 |  | 178 |  | -1.05 |  | 0.295 |  |
| MH Score |  | MH Score |  | -0.01 |  | 0.00 |  | -0.01 |  | -0.01 |  | -0.61 |  | 178 |  | -14.30 |  | < .001 |  |
| MH Score² |  | MH Score² |  | -0.00 |  | 0.00 |  | -0.00 |  | -0.00 |  | -0.72 |  | 178 |  | -10.29 |  | < .001 |  |

# Supplementary Table 3. Regression estimates for polynomial function of Humanness on MH Score and Exposure Condition.

| Polynomial Model Coefficients: Humanness | | | | | | | | | | | | | | | | | | | |
| --- | --- | --- | --- | --- | --- | --- | --- | --- | --- | --- | --- | --- | --- | --- | --- | --- | --- | --- | --- |
|  | | | | | | | | **95% Confidence Interval** | | | |  | | | | | | | |
| **Names** | | **Effect** | | **Estimate** | | **SE** | | **Lower** | | **Upper** | | **β** | | ***df*** | | ***t*** | | ***p*** | |
| Intercept |  | Intercept |  | 2.66 |  | 0.11 |  | 2.45 |  | 2.87 |  | 0.00 |  | 178 |  | 25.09 |  | < .001 |  |
| Condition |  | Long - Brief |  | -0.06 |  | 0.11 |  | -0.27 |  | 0.16 |  | -0.05 |  | 178 |  | -0.52 |  | 0.602 |  |
| MH Score |  | MH Score |  | 0.01 |  | 0.00 |  | 0.01 |  | 0.01 |  | 0.67 |  | 178 |  | 14.32 |  | < .001 |  |
| MH Score² |  | MH Score² |  | 0.00 |  | 0.00 |  | 0.00 |  | 0.00 |  | 0.49 |  | 178 |  | 6.44 |  | < .001 |  |

**Supplementary Table 4.** Descriptive statistics of main variables by group (Experiment 2, between-subjects).

|  | | **Condition** | | **Response Variance** | | **Response Accuracy** | |
| --- | --- | --- | --- | --- | --- | --- | --- |
| Mean |  | Brief |  | 0.08 |  | 0.87 |  |
|  |  | Long |  | 0.06 |  | 0.91 |  |
| Standard deviation |  | Brief |  | 0.10 |  | 0.20 |  |
|  |  | Long |  | 0.09 |  | 0.16 |  |
| Skewness |  | Brief |  | 0.76 |  | -1.77 |  |
|  |  | Long |  | 1.14 |  | -2.08 |  |
| Kurtosis |  | Brief |  | -1.08 |  | 3.10 |  |
|  |  | Long |  | -0.29 |  | 4.03 |  |
| Shapiro-Wilk W |  | Brief |  | 0.76 *** |  | 0.72 *** |  |
|  |  | Long |  | 0.69 *** |  | 0.64 *** |  |
| Note. n = 36. N = 91. * p < .05, ** p < .01, *** p < .001 | | | | | | | |

**Supplementary Table 5.** Kendall’s Tau correlation of main variables (Experiment 2, between-subjects).

|  |  | | **Response Variance** | | **Response Accuracy** | | | **Mechano-Humanness Score** |
| --- | --- | --- | --- | --- | --- | --- | --- | --- |
| Response Variance | | Kendall’s Tau B | | — | |  |  | |
|  |  | p-value | | — | |  |  | |
| Response Accuracy | | Kendall’s Tau B | | -0.97 *** | | — |  | |
|  |  | p-value | | < .001 | | — |  | |
| Mechano-Humanness Score | | Kendall’s Tau B | | 0.42 *** | | -0.42*** | — | |
|  |  | p-value | | < .001 | | < .001 | — | |

Note. n = 36. N = 91. * p < .05, ** p < .01, *** p < .001

# Supplementary Table 6. Regression estimates for polynomial function of Response Variance on MH Score and Exposure Condition (between-subjects).

| \| Polynomial Model Coefficients: Response Variance (between-subjects) \| \| \| \| \| \| \| \| \| \| \| \| \| \| \| \| \| \| \| \| \| --- \| --- \| --- \| --- \| --- \| --- \| --- \| --- \| --- \| --- \| --- \| --- \| --- \| --- \| --- \| --- \| --- \| --- \| --- \| --- \| \|  \| \| \| \| \| \| \| \| **95% Confidence Interval** \| \| \| \|  \| \| \| \| \| \| \| \| \| **Names** \| \| **Effect** \| \| **Estimate** \| \| **SE** \| \| **Lower** \| \| **Upper** \| \| **β** \| \| ***df*** \| \| ***t*** \| \| ***p*** \| \| \| Intercept \|  \| Intercept \|  \| 0.08 \|  \| 0.01 \|  \| 0.06 \|  \| 0.10 \|  \| 0.00 \|  \| 175 \|  \| 8.15 \|  \| < .001 \|  \| \| Condition \|  \| Long - Brief \|  \| -0.02 \|  \| 0.01 \|  \| -0.03 \|  \| -0.01 \|  \| -0.21 \|  \| 175 \|  \| -2.64 \|  \| 0.009 \|  \| \| MH Score \|  \| MH Score \|  \| 0.00 \|  \| 0.00 \|  \| 0.00 \|  \| 0.00 \|  \| 2.43 \|  \| 175 \|  \| 9.88 \|  \| < .001 \|  \| \| MH Score² \|  \| MH Score² \|  \| 0.00 \|  \| 0.00 \|  \| 0.00 \|  \| 0.00 \|  \| 0.68 \|  \| 175 \|  \| 2.62 \|  \| 0.010 \|  \| \| MH Score³ \|  \| MH Score³ \|  \| -0.00 \|  \| 0.00 \|  \| -0.00 \|  \| -0.00 \|  \| -1.82 \|  \| 175 \|  \| -4.40 \|  \| < .001 \|  \| \| MH Score⁴ \|  \| MH Score⁴ \|  \| -0.00 \|  \| 0.00 \|  \| -0.00 \|  \| -0.00 \|  \| -0.54 \|  \| 175 \|  \| -3.84 \|  \| < .001 \|  \| \| MH Score⁵ \|  \| MH Score⁵ \|  \| 0.00 \|  \| 0.00 \|  \| 0.00 \|  \| 0.00 \|  \| 0.37 \|  \| 175 \|  \| 2.21 \|  \| 0.028 \|  \|   Note. Unlike the within-subjects response variance data whose model is significant up to the 4^th^ term, this model is significant up to the 5^th^ term. |
| --- | --- | --- | --- | --- | --- | --- | --- | --- | --- | --- | --- | --- | --- | --- | --- | --- | --- | --- | --- | --- | --- | --- | --- | --- | --- | --- | --- | --- | --- | --- | --- | --- | --- | --- | --- | --- | --- | --- | --- | --- | --- | --- | --- | --- | --- | --- | --- | --- | --- | --- | --- | --- | --- | --- | --- | --- | --- | --- | --- | --- | --- | --- | --- | --- | --- | --- | --- | --- | --- | --- | --- | --- | --- | --- | --- | --- | --- | --- | --- | --- | --- | --- | --- | --- | --- | --- | --- | --- | --- | --- | --- | --- | --- | --- | --- | --- | --- | --- | --- | --- | --- | --- | --- | --- | --- | --- | --- | --- | --- | --- | --- | --- | --- | --- | --- | --- | --- | --- | --- | --- | --- | --- | --- | --- | --- | --- | --- | --- | --- | --- | --- | --- | --- | --- | --- | --- | --- | --- | --- | --- | --- | --- | --- | --- | --- | --- | --- | --- | --- | --- | --- | --- | --- | --- | --- | --- | --- | --- | --- | --- | --- | --- | --- | --- | --- | --- | --- | --- | --- | --- | --- | --- | --- | --- | --- | --- | --- | --- | --- | --- | --- | --- | --- | --- | --- | --- | --- | --- | --- | --- | --- | --- | --- | --- | --- | --- | --- | --- | --- | --- |

**Supplementary Table 7.** Regression estimates for polynomial function of Response Accuracy on MH Score and Exposure Condition (between-subjects).

| Polynomial Model Coefficients: Response Accuracy (between-subjects) | | | | | | | | | | | | | | | | | | | |
| --- | --- | --- | --- | --- | --- | --- | --- | --- | --- | --- | --- | --- | --- | --- | --- | --- | --- | --- | --- |
|  | | | | | | | | **95% Confidence Interval** | | | |  | | | | | | | |
| **Names** | | **Effect** | | **Estimate** | | **SE** | | **Lower** | | **Upper** | | **β** | | ***df*** | | ***t*** | | ***p*** | |
| Intercept |  | Intercept |  | 0.88 |  | 0.02 |  | 0.84 |  | 0.92 |  | 0.00 |  | 176 |  | 39.82 |  | < .001 |  |
| Condition |  | Long - Brief |  | 0.04 |  | 0.02 |  | 0.01 |  | 0.07 |  | 0.22 |  | 176 |  | 2.36 |  | 0.019 |  |
| MH Score |  | MH Score |  | -0.00 |  | 0.00 |  | -0.01 |  | -0.00 |  | -1.83 |  | 176 |  | -11.68 |  | < .001 |  |
| MH Score² |  | MH Score² |  | -0.00 |  | 0.00 |  | -0.00 |  | -0.00 |  | -0.79 |  | 176 |  | -2.73 |  | 0.007 |  |
| MH Score³ |  | MH Score³ |  | 0.00 |  | 0.00 |  | 0.00 |  | 0.00 |  | 0.89 |  | 176 |  | 7.53 |  | < .001 |  |
| MH Score⁴ |  | MH Score⁴ |  | 0.00 |  | 0.00 |  | 0.00 |  | 0.00 |  | 0.54 |  | 176 |  | 3.64 |  | < .001 |  |

**Supplementary Table 8.** Regression estimates for polynomial function of Response Variance on MH Score and Exposure Condition (within-subjects).

| Polynomial Model Coefficients: Response Variance (within-subjects) | | | | | | | | | | | | | | | | | | | |
| --- | --- | --- | --- | --- | --- | --- | --- | --- | --- | --- | --- | --- | --- | --- | --- | --- | --- | --- | --- |
|  | | | | | | | | **95% Confidence Interval** | | | |  | | | | | | | |
| **Names** | | **Effect** | | **Estimate** | | **SE** | | **Lower** | | **Upper** | | **β** | | **df** | | **t** | | **p** | |
| Intercept |  | Intercept |  | 0.07 |  | 0.01 |  | 0.05 |  | 0.09 |  | 0.00 |  | 176 |  | 8.24 |  | < .001 |  |
| Condition |  | Long - Brief |  | -0.03 |  | 0.01 |  | -0.05 |  | -0.02 |  | -0.37 |  | 176 |  | -5.26 |  | < .001 |  |
| MH Score |  | MH Score |  | 0.00 |  | 0.00 |  | 0.00 |  | 0.00 |  | 1.99 |  | 176 |  | 17.27 |  | < .001 |  |
| MH Score² |  | MH Score² |  | 0.00 |  | 0.00 |  | 0.00 |  | 0.00 |  | 0.93 |  | 176 |  | 4.41 |  | < .001 |  |
| MH Score³ |  | MH Score³ |  | -0.00 |  | 0.00 |  | -0.00 |  | -0.00 |  | -0.88 |  | 176 |  | -10.25 |  | < .001 |  |
| MH Score⁴ |  | MH Score⁴ |  | -0.00 |  | 0.00 |  | -0.00 |  | -0.00 |  | -0.56 |  | 176 |  | -5.18 |  | < .001 |  |
|  | | | | | | | | | | | | | | | | | | | |

# Supplementary Table 9. Regression estimates for polynomial function of Response Variance on MH Score and Exposure Condition (within-subjects).

| Polynomial Model Coefficients: Response Accuracy (within-subjects) | | | | | | | | | | | | | | | | | | | |
| --- | --- | --- | --- | --- | --- | --- | --- | --- | --- | --- | --- | --- | --- | --- | --- | --- | --- | --- | --- |
|  | | | | | | | | **95% Confidence Interval** | | | |  | | | | | | | |
| **Names** | | **Effect** | | **Estimate** | | **SE** | | **Lower** | | **Upper** | | **β** | | **df** | | **t** | | **p** | |
| (Intercept) |  | (Intercept) |  | 0.89 |  | 0.02 |  | 0.85 |  | 0.94 |  | 0.00 |  | 176 |  | 42.81 |  | < .001 |  |
| Condition1 |  | Long - Brief |  | 0.05 |  | 0.02 |  | 0.02 |  | 0.08 |  | 0.27 |  | 176 |  | 3.02 |  | 0.003 |  |
| MH Score |  | MH Score |  | -0.01 |  | 0.00 |  | -0.01 |  | -0.00 |  | -1.86 |  | 176 |  | -12.63 |  | < .001 |  |
| MH Score² |  | MH Score² |  | -0.00 |  | 0.00 |  | -0.00 |  | -0.00 |  | -1.07 |  | 176 |  | -3.93 |  | < .001 |  |
| MH Score³ |  | MH Score³ |  | 0.00 |  | 0.00 |  | 0.00 |  | 0.00 |  | 0.86 |  | 176 |  | 7.78 |  | < .001 |  |
| MH Score⁴ |  | MH Score⁴ |  | 0.00 |  | 0.00 |  | 0.00 |  | 0.00 |  | 0.64 |  | 176 |  | 4.59 |  | < .001 |  |
|  | | | | | | | | | | | | | | | | | | | |
